# Attachment Preference in Auditory German Sentences: Individual Differences and Pragmatic Strategy

**DOI:** 10.3389/fpsyg.2019.01357

**Published:** 2019-06-18

**Authors:** Eleanor E. Harding, Daniela Sammler, Sonja A. Kotz

**Affiliations:** ^1^Department of Neuropsychology, Max Planck Institute for Human Cognitive and Brain Sciences, Leipzig, Germany; ^2^Otto Hahn Group “Neural Bases of Intonation in Speech and Music”, Max Planck Institute for Human Cognitive and Brain Sciences, Leipzig, Germany; ^3^Department of Neuropsychology and Psychopharmacology, Faculty of Psychology and Neuroscience, Maastricht University, Maastricht, Netherlands

**Keywords:** attachment preference, individual differences, pragmatics, musical ability, working memory, syntax

## Abstract

Relative clauses modify a preceding element, but as this element can be flexibly located, the point of attachment is sometimes ambiguous. Preference for this attachment can vary within languages such as German, yet explanations for differences in attachment preference related to cognitive strategies or constraints have been conflicting in the current literature. The present study aimed to assess the preference for relative clause attachment among German listeners and whether these preferences could be explained by strategy or individual differences in working memory or musical rhythm ability. We performed a sentence completion experiment, conducted *post hoc* interviews, and measured working memory and rhythm abilities with diagnostic tests. German listeners had no homogeneous attachment preference, although participants consistently completed individual sentences across trials according to the general preference that they reported offline. Differences in attachment preference were moreover not linked to individual differences in either working memory or musical rhythm ability. However, the pragmatic content of individual sentences sometimes overrode the general syntactic preference in participants with lower rhythm ability. Our study makes an important contribution to the field of psycholinguistics by validating offline self-reports as a reliable diagnostic for an individual’s online relative clause attachment preference. The link between pragmatic strategy and rhythm ability is an interesting direction for future research.

## Introduction

Ambiguous relative clause attachment—a modification that could be plausibly attached to more than one preceding linguistic element—is a standing cross-linguistic enigma (see Sanz et al., 2013), and the field of psycholinguistics can benefit by explaining variability in the processing of attachment ambiguity during comprehension. However, a caveat for such investigations is that native speakers of the same language can have different preferences for the general “naturalness” of the same syntactic attachments (e.g., Augurzky, 2006). A large portion of related investigation into the underlying causes of individual differences in attachment preferences has focused on working memory capacity, but has produced mixed results across the literature (Hocking, 2003; Swets et al., 2007; Traxler, 2007; Caplan and Waters, 2013). Interestingly, recent decades have produced observations linking musical rhythm skills with language comprehension (e.g., Tierney and Kraus, 2014; Gordon et al., 2015a), suggesting the usefulness of including a musical rhythm metric along with a traditional working memory metric when investigating the processing of ambiguous syntactic attachment. The present sentence-completion study therefore had two aims. First, to establish a reliable way to determine individual native-speaker preference for ambiguous syntactic attachment, for use in future psycholinguistic studies about syntactic ambiguity comprehension. Second, to test the explanatory power of working memory and rhythm ability where differences in attachment preferences occurred.

(1) Das sind die Freunde der Chefinnen, die Ghent vor kurzem besuchten.
  *There are the friends of the bosses, who recently visited Ghent*.

Relative clauses modify or describe a linguistic element such as a noun or another clause and are introduced by relative pronouns such as *who* or *which.* In sentences such as (1), the relative clause can be ambiguously attached, meaning it could either attach high to the first noun phrase (NP1) “the friends” or low to the second noun phrase (NP2) “the bosses.” Readers or listeners typically disambiguate attachment ambiguities by means of prosody (e.g., Jun, 2010). Prosody, an arm of phonology, is concerned with meaningful perceptual units that are defined by phonological and phonemic aspects such as pitch intonation, lengthening, and loudness (Nespor and Vogel, 1986), features attributed to the “rhythm” of speech (e.g., Kotz et al., 2018). Readers and listeners typically have a preference for high or low attachment that is informed by implicit or default prosody in silent reading or unbiased naïve production (Fodor, 1998, 2002; Jun, 2010).

In German, the language of the present study, observations of high and low attachment preference of native readers and listeners have been mixed (Hemforth et al., 2000, 2015; Augurzky, 2006). A particular sentence construction such as (1) is particularly relevant to the study’s two aims: A two-clause sentence, wherein the main clause contains two noun phrases (NP1 and NP2) such that NP2 is in the genitive case, possessed by NP1, and a relative clause attaches ambiguously to NP1 or NP2. In Augurzky (2006), German sentences with this type of construction were shown to tax comprehension processes more than other types of NP1–NP2 relationships (e.g., connected by various prepositions), making them suitable to study individual attachment preference (our aim 1). Moreover, as long as overt prosody did not bias low attachment, sentences such as (1) yielded a heterogeneous attachment preference across items and participants (Augurzky, 2006), making it an ideal construction to elucidate explanatory factors for these differences (our aim 2).

Multiple syntactic parsing accounts attribute attachment preference to working memory capacity (Baddeley and Hitch, 1974), intuitively assuming that longer and more complex high-attachment structures require more working memory (e.g., Frazier, 1978; Just and Carpenter, 1992; Gibson, 2000). However, no clear role can be gleaned from incompatible observations of its role in attachment preference across the literature (Hocking, 2003; Swets et al., 2007; Traxler, 2007; Caplan and Waters, 2013; cf. Jun, 2010, for an interpretation of mixed results). Other strategies that may determine attachment preference are thematic (the roles adopted by interacting words in a sentence; De Vincenzi and Job, 1995) or pragmatic categories (real-world knowledge; Scheepers, 2003). In order to address these reported strategies in attachment preference, the current study employed the modified listening span (MLS) (Daneman and Carpenter, 1980), used as a working memory metric in previous attachment-preference experiments (e.g., Swets et al., 2007; Traxler, 2007), and conducted a *post hoc* interview wherein participants were asked to describe how they chose to complete the experimental sentence trials. In a novel approach, we also adopted a second cognitive measure in a different, non-verbal domain: music.

Comprehending ambiguous syntactic structures relates to musical rhythm skills, as follows: Syntactic attachment cues are guided by prosody (e.g., Clifton et al., 2002; Augurzky, 2006; Jun, 2010), in particular acoustic cues delivered by phonological constituents (Nespor and Vogel, 1986). Empirical evidence supports that musical rhythm ability positively impacts phonological processing (e.g., Tierney and Kraus, 2014; Gordon et al., 2015b). Improved phonological processing linked to better rhythm skills has further been attributed to improvements in language (reading) comprehension (Douglas and Willatts, 1994; Gordon et al., 2015a; cf. Swaminathan et al., 2018). Therefore, we included a musical rhythm abilities test (Wallentin et al., 2010) when investigating the cognitive architecture salient to relative clause attachment.

Thus, the current experiment explored auditory relative clause attachment preference in German, potential underlying strategies for ambiguity resolution, and the possible roles of verbal working memory and musical rhythm ability. Participants were a group of monolingual native German speakers. Consistent with previous auditory domain reports (Augurzky, 2006), we found that attachment preference varied between individuals but that participants reliably reported their own preferences. While verbal working memory had no impact on attachment preference, we found that rhythm ability correlated with a pragmatic ambiguity resolution strategy that was discovered in the *post hoc* interviews.

## Materials and Methods

### Participants

Thirty monolingual native German speakers (14 males), aged between 21 and 34 years (*M* = 25.2 years, *SD* = 3.12) took part in the study at the Max Planck Institute for Human Cognitive and Brain Sciences in Leipzig, Germany. Participants reported their (and their parents’) place of birth and whether they were raised speaking formal German or a colloquial dialect. All participants were paid 7€ per hour and provided informed consent. The study was approved by the local ethics committee of the University of Leipzig.

### Materials

#### Stimuli

**FIGURE 1:**
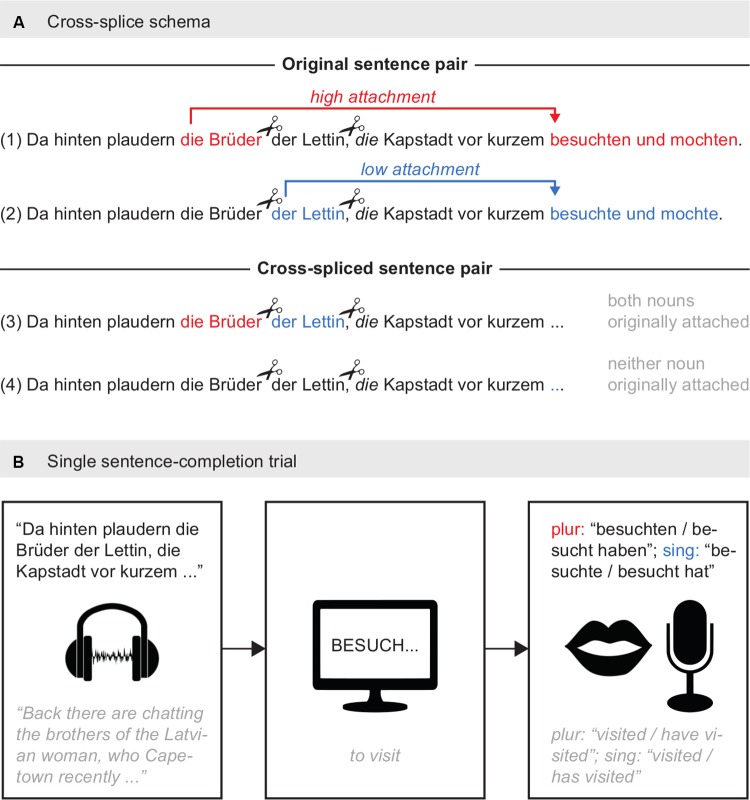
Paradigm and cross-splice schema. **(A)** Full sentences were recorded in pairs, each with a resolved high attachment (1) and low attachment (2) of the relative clause. Sentences were cross-spliced to neutralize the impact of potential implicit prosodic cues from the speaker. The final cross-spliced stimulus pairs ended before the final verbs, and contained either both nouns (3) or neither noun (4) that were originally attached to the relative clause. The final sentence-pair versions were counterbalanced across participants, so that each participant only heard the lexical combination once. **(B)** Participants heard a sentence fragment containing an ambiguous relative clause and were instructed to complete the sentence with their preferred singular or plural conjugation of the word “*besuchen*”*/*“*to visit.*” A singular verb would attach low, and a plural verb would attach high.

One hundred twenty sentence pairs with ambiguously attached relative clauses were composed by a native German speaker. Sentences consisted of a main clause and a relative clause. In the main clause, the plural subject (NP1) possessed a genitive object (NP2), and the immediately following relative pronoun was ambiguously attached to NP1 or NP2. The relative clause attachment was disambiguated with a plural (high attachment) or singular (low attachment) conjugation of the final verb (Figure 1A) in each pair. The sentence construction closely followed stimuli used in Augurzky (2006), where neutral prosody yielded mixed attachment preference in German participants, and both attachment sites were difficult to comprehend. Thus, this syntax was ideally suited for the current investigation of individual differences in attachment preference, for future application in sentence comprehension research.

The lexical content of the sentences was strictly controlled. Two prepositions started each sentence (“*Da hinten*”*/*“*Back there*”; 10 combinations), followed by a verb conjugated for the first plural noun (“*plaudern*”*/*“*are chatting*”; 10 verbs total), then an article with plural noun (“*die Brüder*”*/*“*the brothers*”; 15 animate words of relation: aunts, friends, etc.) to set up the first possibility for the ambiguous relative pronoun, next a genitive article and singular noun (“*der Lettin*”*/*“*of the Latvian woman*”; 15 animate words of a nationality, title or profession: heiress, customer, English woman, etc.), providing the second option for the ambiguous relative pronoun. In the second clause, the first item was always “die”/who, followed by a city (“*Kapstadt*”*/*“*Capetown*”; 30 locations, either well-known international cities or cities within Germany), followed next by the verbs “besuchen” and “mögen”/to visit and to like (conjugated for either singular: “*besuchte und mochte*,” or plural: “*besuchten und mochten*”*/*“*visited and liked*”). Lexical items were pseudo-randomized to create 120 sentence versions (Appendix 1, Supplementary Data Sheet 3 in Supplementary Material). The lexical items for the sentences were controlled for frequency using the Leipziger Wortschatz and Celex word databases.

##### Recording and digitization

A trained female native German speaker spoke the sentences in a soundproof room. She was given time to read and comprehend the sentences before production.

Spoken stimuli similar to ours, with the genitive NP1–NP2—relative clause construction, previously yielded heterogeneous attachment preference for both neutral- and high-attachment prosody—essentially as long as the speaker did not overtly bias low attachment (Augurzky, 2006). Therefore, the current speaker was instructed not to assign any special prosodic intonation to either of the nouns to which the ambiguous relative pronoun referred, which could generate an unwanted context for low-attachment bias (e.g., Schafer et al., 1996).

Moreover, neutral or unbiased production for this genitive construction previously followed high-attachment prosody (Augurzky, 2006), which is based primarily on lengthening cues, with a larger pause before the relative clause than between the NPs (e.g., Clifton et al., 2002; Jun, 2010). Therefore, the current speaker received no instructions in terms of prosodic lengthening cues, thus following the “default” high-attachment prosody necessary for our paradigm (Clifton et al., 2002; Augurzky, 2006; Jun, 2010).

Sentences were recorded with a Rode NT55 microphone (Silverwater, Australia) at 16-bit resolution and a sampling rate of 44.1 kHz using Cool Edit Pro 2.0 (Sibiu, Romania). After the sentences were recorded, they were normalized to 70 dB and cross-spliced, and the final verb was cropped for use in the sentence completion task using Praat 5.2 (Boersma and Weenink, 2011).

##### Cross-splicing

Although the speaker was instructed not to give any prosodically disambiguating intonation to the subject in the main clause, sentences were post-processed to rule out any subconscious cuing by the speaker. The resulting cross-splicing plan exchanged critical words in the noun phrase from the first clause (Figure 1A). The NP1 and NP2 always retained original order. All cross-splicing preserved the lengthening of NP1, NP2, and the subsequent comma, reported to be the most important cues for high-attachment prosody (e.g., Jun, 2010). After the cross-splicing, sentences within the pair would have either both nouns that were originally attached to the relative clause (“hosting”) or both nouns that were originally not attached (“not hosting”). The final disambiguation of the relative clause, i.e., the verbs, was cropped. The prosodic naturalness of the cross-spliced stimuli was informally evaluated, and approved, by eight native German speakers who had some background in linguistics. Participants were presented with each lexical combination only once (120 items per participant), and the items (240 total) were balanced to equally represent pre-critical-words in both syntactic conditions. Final stimuli .wav files may be found in Supplementary Material.

#### Diagnostic Tests

The current paradigm offered an opportunity to assess individual differences in cognitive resources related to relative clause attachment in the auditory domain. In the face of conflicting working memory evidence (cf. Hocking, 2003; Swets et al., 2007; Traxler, 2007), a musical skills metric (MET; Wallentin et al., 2010) was introduced in addition to the traditional working memory diagnostic test (MLS, adapted from Daneman and Carpenter, 1980).

##### Modified listening span

The MLS is the auditory version of the modified reading span (MRS; Daneman and Carpenter, 1980), which has been widely used in attachment preference literature as a working memory capacity metric (e.g., Hocking, 2003; Swets et al., 2007; Traxler, 2007). The MLS/MRS was designed to index the trade-off between storage and processing in the working memory system, specifically as it pertains to language comprehension, and correlates with test scores of reading comprehension. In this task, adapted to German from English, participants heard a series of sentences and were instructed to repeat the last word of each sentence, in the order of presentation, once a cross appeared on the screen after the last sentence. Items were spoken by a professionally trained female native German speaker. In addition, participants were instructed to evaluate each sentence for global accuracy (e.g., “All coats are brown” is a globally false statement) and answer “true” or “false” with a timed button response after each sentence. After a training with three sentences, the task began with three blocks of three sentences, and continued up to three blocks of seven sentences or until the participant could no longer perform the task. A level was considered complete if two of the three blocks were answered correctly, and the final score per participant was determined by the highest level he/she completed.

##### Musical ear test rhythm subtest (METr)

This rhythm test developed by Wallentin et al. (2010) employs primarily beat detection and interval discrimination. The original publication distinguished musicianship skills among amateur and professional musicians (Wallentin et al., 2010). During the test, consisting of 52 trials, participants were instructed to listen to rhythm pairs and determine whether the two are the same (“yes” or “no” response button). Half of the trials were “yes” and “no” correct response, presented in randomized order throughout the subtests. Participants had 1 s to respond. The rhythms were beat sequences presented with midi wood-block sounds; difficulty again spanned from easy sequences (rhythms had a few beats with large temporal interval differences between the sequences) to extremely hard (fast, syncopated rhythms with as little as 50-ms difference in temporal intervals between series). Authors reported the subdivision of beats into triplets to be a key feature in difficulty. A faint metronome was played in the background of all melody and rhythm trials, which established the downbeat. A final score up to 52 was assigned per participant.

### Procedure

The auditory sentence completion experiment was conducted in a quiet room. Stimuli (120 items per participant) were presented with PRESENTATION (Version 15.1 ^1^) using Sony MDR-XD100 stereo headphones (Sony Corporation, Tokyo, Japan) adjusted to a comfortable volume. Per trial (Figure 1B), participants heard a sentence fragment that ended before the final verb, and then the verb stem BESUCH—(“to visit”) appeared on the screen. Participants were instructed to listen to incomplete sentences about people who visited cities and to verbally complete the sentence with the most sensible or natural-sounding conjugated form of the verb *besuchen*. Their answer was to be spoken in the flow of the sentence and without reflection. Before beginning the experiment, they completed a short training to test their understanding of the instructions and to assure that their enunciation was clear. Trials were self-paced with a button press. The experimenter logged the answers.

Testing lasted approximately 15 min. *Post hoc*, the experimenter asked participants to consider the sentence fragments they had just completed, and whether it generally seemed more natural (their “gut feeling”) for the first-mentioned people to visit the city or for the second-mentioned person to visit the city. For this response, participants were further instructed to not consider the individual items such as the actual descriptions of the people, nor which city, nor to factor in their actual sentence completion responses. They were assured that the general preference did not have to reflect how many sentences they completed according to the preference. Based on these answers, participants were assigned general offline preference for relative clause attachment—high-attachment preference for participants who thought it sounded more natural for the first-mentioned people (NP1) to visit the city, and low-attachment preference for participants who thought it sounded more natural to have the second-mentioned person (NP2) visit the city. A semi-structured interview followed. During the interview, participants were asked to explain in detail what motivated their sentence completion answers, and the experimenter directed follow-up questions to their offline attachment preference (e.g., “You said that you prefer the second-mentioned person to visit the city in general, but that you completed many sentences with the first-mentioned people visiting the city. What was it about those sentences that made you go against your general preference?”). Participants were further asked their and their parents’ geographic origin and use of local dialect and High German. Finally, participants were invited to share any general comments about the experiment or stimuli. The experimental session (sentence completion and interview) was recorded with a Zoom H4n Handy recorder (2009).

After the experiment and interview, participants completed the MLS (Daneman and Carpenter, 1980) and the METr (Wallentin et al., 2010). Instructions were written. Computerized versions of the diagnostic tests were administered to participants at a comfortable volume *via* headphones. Correct-hand response was counterbalanced across participants but kept consistent for tests within participant. The order of diagnostic test presentation was pseudo-randomized.

### Analysis

Non-parametric tests were used in all analyses as testing measures either were not normally distributed, had unequal sizes when comparing groups, or yielded non-normally distributed residuals from parametric analyses.

Recordings from the experimental session were consulted for the analysis. For each trial, relative clause attachment was determined from the verb conjugation spoken by participants: plural conjugation indicated a high attachment while singular conjugation indicated a low attachment. Across trials, participants answered spontaneously in either simple past tense or perfect tense (Figure 1B). Both are valid syntactic constructions in German and were treated equivalently in the analysis. Acceptable simple past answers “*besuchten*” (plur). or “*besuchte*” (sing). provided the critical morpheme as the final syllable “*-ten*”*/*”*-te*” in the verb. Acceptable perfect tense answers “*besucht haben/hatten*” (plur). or “*besucht hat/hatte*” (sing). provided the present or past participle along with a conjugated form of an auxiliary verb such as *haben* “to have.”

#### Online Attachment Preference (Sentence Completion Answers)

Sentence completion answers were analyzed both by-subject and by-item. Scores were derived from treating answers as a proportion of how many were attached high, e.g., items or subjects with a score of 1 were always high, those with a score of 0 were always low, and those with a score of 0.5 had a perfect split between high and low attachment.

In order to see whether participants reliably perceived their own attachment preference, sentence completion scores were assessed in terms of an offline self-report of attachment preference. In order to find any possible links between attachment preference and cognitive mechanisms, sentence completion scores by subject were assessed in terms of strategies, working memory, and musical rhythm ability. These analyses are described in the sections “Offline Attachment Preference (Participant Self-Reports),” “Strategy,” and “Working Memory and Musical Rhythm Ability.”

#### Offline Attachment Preference (Participant Self-Reports)

One aim of the study was to find a reliable way to determine an individual’s attachment preference, in order to aid future psycholinguistic comprehension studies. In a first step toward this aim, after completion of the experiment, participants were asked to reflect on the sentence fragments and answer whether any one type of verb conjugation (singular or plural) seemed to systematically sound most natural (see the section “Procedure”). Considering the distance between the actual answer and the judgment, this self-report was referred to as the “offline” attachment preference. The offline attachment preference was assigned “high” if participants reported preferred plural conjugations and “low” if participants reported preferred singular conjugations. Six participants who reported either not having a preference or switching their preference during the course of the experiment were excluded (final *n* = 24).

In a second step toward our aim of reliably assigning participant attachment preference, the sentence completion answers were evaluated to see whether they appropriately fell into the offline attachment preference category that participants reported. To do so, the by-subject averages were compared between the group of participants who reported an offline high-attachment preference and the group who reported offline low-attachment preference. As sentence completion answers were non-normally distributed, and moreover as offline attachment preference groups were of unequal sizes, the means had to be compared with a non-parametric test. Mann–Whitney’s *U*, an alternative to the parametric *t*-test, ranked the sentence completion scores and then compared whether the mean ranks of the groups were significantly different. The null hypothesis was the same as the *t*-test, namely, that mean ranks would not differ between groups.

Finally, in order to see whether the offline reported attachment preferences were systematic across participants, i.e., whether more participants chose low or high as offline preference, the group sizes were tested against chance. The one-sample Wilcoxon rank test, a non-parametric alternative to the one-sample *t*-test, assessed whether the proportion of a group size compared to *N* was significantly different from chance (0.5 probability).

#### Strategy

In order to assess any potential strategies underlying attachment preference, the *post hoc* semi-structured interviews (see the section “Procedure”) were qualitatively evaluated for any patterns pertaining to online or offline attachment preference and geographical origin, usage of dialect versus High German, or the overall motivation for the online answers. No pattern emerged with regard to region of origin or use of dialect or High German. However, many participants brought up real-world knowledge influencing their sentence completion, such as how far away the city being visited was, and which of the two possible nouns (first- or second-mentioned) was more likely to visit it. In other words, these participants described a pragmatics strategy, which has precedence in relative-clause attachment experiments in the psycholinguistics literature (e.g., Scheepers, 2003).

Among participants who brought up real-world knowledge, they reported that it influenced the sentence completion but not the offline reported preference for attachment. For example, one participant reported that it sounded better if the sentences ended with the plural verb—that the first-mentioned people visited the city, but for instances such as sentence (2)

(2) Da hinten laufen die Diener der Ärztin, die Kapstadt vor kurzem ______.
  (literal) Back there are walking the servants of the doctor, who Cape Town recently ______.
  (grammatical) The servants of the doctor, who recently ______ Cape Town, are walking back there.

He completed the sentence with a singular conjugated verb because he assumed a doctor would be richer, more likely to visit Cape Town than servants. By contrast, other participants reported that their “gut feeling” of whether the completion should be a singular or plural verb was strong, and it did not matter what the content of the sentences was.

Quantitative metrics were then established in order to assess this qualitative observation about a reported pragmatics strategy. Therefore, the experimenter assigned participants to a “reported pragmatic strategy” group or a “no reported pragmatic strategy” group based on whether they indicated an influence of real-world knowledge in their semi-structure interviews. Then, online attachment answers were recoded to reflect the proportion of answers that either matched participants’ offline attachment preference or overrode this preference (in favor of pragmatics). For example, in this recoding, 1 would indicate that participants always completed sentences according to their offline attachment preference, 0 would indicate that no sentences were completed according to the offline preference, and 0.5 would indicate that completion answers matched the offline preference in half of the trials.

We wanted to know whether the strategy uncovered in our semi-structured interviews occurred in a significant majority or minority of participants. Therefore, in order to assess whether participants were evenly distributed in their reported use of a pragmatic strategy, a one-sample binomial test, which tests the chance that data fall into one of two binary categories, tested the proportion of participants in each group against 0.5 probability.

We tested whether the reported pragmatic strategy had any statistical relationship to online or offline attachment preference. Online preference: in order to see whether the reported use of a pragmatic strategy coincided with fewer sentence completion answers that matched offline attachment preference, a Mann–Whitney *U* test (see the section “Offline Attachment Preference (Participant Self-Reports)”) compared the mean ranks of the recoded completion answers between the two groups. Offline preference: in order to see whether the reported use of a pragmatic strategy occurred significantly more or less according to the offline reported preference, a Fisher’s exact test evaluated whether offline high- or low-attachment preference was distributed evenly among participants in the two “reported pragmatic strategy” groups. Fisher’s test is similar to the chi-squared distribution; however, it is the better choice for small sample sizes such as ours, where the number of observations in a cell is less than 5.

#### Working Memory and Musical Rhythm Ability

Working memory capacity has theoretical links to syntactic parsing strategies (e.g., Frazier, 1978) and has been previously reported with mixed results in relative clause attachment literature (e.g., Hocking, 2003; Swets et al., 2007). Musical rhythm ability has empirical links to phonological processing (e.g., Tierney and Kraus, 2014), and phonological processing is a component of prosodic processing (e.g., Nespor and Vogel, 1986), which, in turn, is essential to relative clause attachment preference (e.g., Fodor, 1998). Therefore, the MLS (Daneman and Carpenter, 1980) and METr (Wallentin et al., 2010) scores were assessed in terms of their statistical relationship with online and offline relative clause attachment preference. Both scores were correlated with the online proportion of high-attachment answers (Spearman, two-tailed), and Mann–Whitney *U* tests compared both scores between groups of offline-reported high- and low-attachment preference.

## Results

Online attachment preference during the sentence completion experiment was heterogeneous across participants. Despite this heterogeneity, results showed that participants were reliably able to offline-report their own relative clause attachment preference and to reliably assess whether they used a pragmatic strategy. Attachment preference (both online and offline) was unrelated to pragmatic strategy, working memory, and musical rhythm ability. Interestingly, a *post hoc* analysis showed that participants who had low rhythm ability used pragmatic strategy more often than participants with high rhythm ability. Summary data results may be found in Supplementary Material.

### Online Attachment Preference (Sentence Completion Answers)

Results by subject in the online sentence completion experiment showed heterogeneous attachment preferences across participants. This was indicated by a trimodal distribution of the proportion of sentences completed with high attachment. The distribution was skewed right (0.35), with a central peak near 0.5 and outer peaks near 0 and 1. The mean, 0.41 (*SD* = 0.31), was not different from chance (one-sample Wilcoxon ranks test against a 0.5 median; *p* = 0.200).

Results by item (*N* = 240) showed a mean score of 0.48 (1 being only high-attachment answers, 0 being only low-attachment answers). The confidence interval was 0.46 to 0.49; median = 0.47; *SD* = 0.12; *SE* = 0.008, range = 0.13–0.87. The distribution appeared normal; however, likely due to the high *N* and very low *SE*, the distribution did not pass a Shapiro–Wilk test of normality (statistic = 0.97, *p* < 0.0001). Likewise, in a non-parametric one-sample Wilcoxon ranks test against a 0.5 median, the items were significantly less than 0.5 (*p* = 0.004). This was likely a Type I error, again due to the low SE. The one-sample Wilcoxon ranks test was run again with a 0.49 chance level, and this time, it was not significant (*p* = 0.347).

An additional by-item analysis was performed to rule out any effects of the cross-splicing on online attachment preference. Stimulus items with “hosting” versus “not hosting” cross-splice versions (see the section “Cross-splicing”) were compared with a two-independent-samples Mann–Whitney test. There was no significant difference between the medians of items originally both “hosting” (mean rank = 119.64) versus “not-hosting” (mean rank = 121.36; Mann–Whitney *U* = 7096.5, *p* = 0.845). Moreover, during the interviews, no participants remarked that the sentences sounded unnatural. Thus, the cross-splicing had no apparent bearing on the attachment answers.

### Offline Attachment Preference (Participant Self-Reports)

**FIGURE 2:**
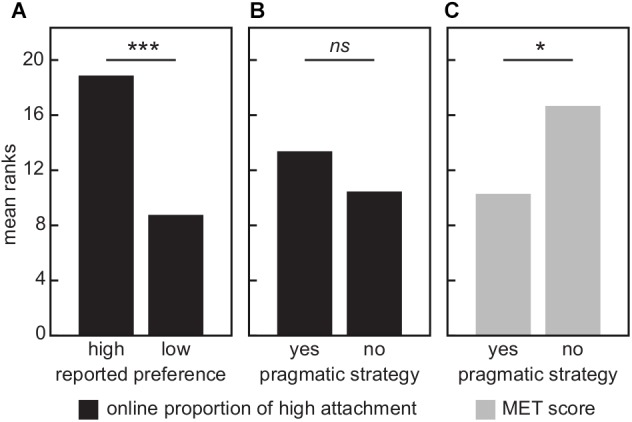
Group comparisons. **(A)** Online proportion of high attachments significantly differed between participants with offline reported high- and low-attachment preferences. **(B)** Use of a pragmatic strategy (yes/no) did not influencethe overall online proportion of high attachments. **(C)** *Post hoc* observation that participants who resorted to pragmatic strategy had lower musical ability (MET score; Wallentin et al., 2010). All scores were converted to mean ranks for Mann–Whitney *U* tests. ^∗^*p* < 0.05 and ^∗∗∗^*p* < 0.001.

Reported preference was heterogeneous for high or low relative clause attachment. A majority of participants reported a preference for low attachment (15 out of 24), but this majority was not significantly different from chance (one-sample binomial test against 0.5 probability; *p* = 0.300). Listeners’ reported attachment preference matched their actual sentence completion answers (Figure 2A). The group with reported high-attachment preference (*n* = 9) had a significantly higher proportion of high-attachment answers (mean rank = 18.9) than the group with reported low-attachment preference (*n* = 15, mean rank = 8.8; *U* = 10.0, *p* = 0.001). Importantly, these results validate a psycholinguistics approach that factors in an individual participants’ self-reported attachment preference when testing comprehension of ambiguous relative clauses, which can contribute to future studies.

### Strategy

While some participants (*n* = 8) completed trial sentences based solely on their offline general attachment preference, other participants (*n* = 16) reported that they sometimes attached relative clauses based on the pragmatic context of the sentence (e.g., servants were less likely to visit South Africa than a doctor), even when the pragmatic answer went against their attachment preference and seemed structurally less natural. The presence or absence of this pragmatic strategy was randomly distributed across participants (one-sample binomial test, *p* = 0.200).

Pragmatic strategy significantly influenced the proportion of answers that matched participants’ offline attachment preference, verifying participants’ own perception whether they were influenced by pragmatics. The group without a pragmatic strategy had a significantly higher proportion of attachment answers that matched their reported preference (mean rank = 20.5) than the group with a pragmatic strategy (mean rank = 8.5; *U* = 0.0, *p* < 0.001).

While pragmatic strategy affected how often participants went against their own attachment preference on a trial-by-trial basis, the presence or absence of a pragmatic strategy had no statistical relationship to actual preference for high or low relative clause attachment (Figure 2B). This was the case for both the offline general structural preference (Fisher’s test with two self-reported measures, *p* = 1) and the online, trial-by-trial proportion of high-attached completed sentences (mean rank reported strategy = 13.5; mean rank no reported strategy = 10.5; *U* = 48.0, *p* = 0.300; Figure 2B).

### Working Memory and Musical Rhythm Ability

The working memory and musical rhythm ability metrics were used to assess individual differences in relative clause attachment preference, but had no statistical relationship with attachment preference. Neither MLS (Daneman and Carpenter, 1980) scores nor METr (Wallentin et al., 2010) scores differed according to offline reported high-attachment (mean rank MLS = 11.2, METr = 13.0) or low-attachment preference (mean rank: MLS = 13.3, METr = 12.2) (Mann–Whitney *U:* MLS = 56.0, METr = 63.0, *p*’s > 0.5). Neither score correlated with the online proportion of sentences completed with high attachment (Spearman’s, MLS rho = −0.24, *p* = 0.300; METr rho = −0.03, *p* = 0.900).

### *Post hoc* Analysis: Pragmatic Strategy and Musical Ability

During the interviews, the experimenter observed that several participants performed particularly poorly on the METr and that these participants, in turn, reported real-world knowledge to be a large influence on their sentence completion answers. In one case, this was even accompanied by a participant’s difficulty in comprehending the notion of syntax or discussing the sentences in terms of structure—he relied exclusively on pragmatic information to inform his sentence completions. In order to follow up this observation, METr scores were compared between the “reported pragmatic strategy” and “no reported pragmatic strategy” groups in a *post hoc* Mann–Whitney *U* test analysis (see the sections “Offline Attachment Preference (Participant Self-Reports)” and “Strategy”). Indeed, mean-ranked METr scores were significantly lower among participants who reported using a pragmatic strategy (mean rank = 10.1) compared to those who did not report using a pragmatic strategy (mean rank = 17.2, *U* = 26.0, *p* = 0.019; Figure 2C).

## Discussion

The current study aimed to assess the preference for relative clause attachment among German listeners, and whether those preferences could be explained by strategy or individual differences in working memory or musical rhythm ability. While German listeners had no homogeneous attachment preference, participants consistently completed individual sentences across trials according to the general preference that they reported offline. These differences in attachment preference were not linked to individual differences in either working memory or rhythm ability. However, the pragmatic content of individual sentences sometimes overrode the general syntactic preference in participants with lower rhythm ability.

The main goal of this study was to establish a reliable way to account for individual differences in relative clause attachment preference. Although a non-significant majority preferred low attachment, the heterogeneous attachment preference was replicated here. Importantly, participants’ online attachment preference reflected their offline reported general preference for high- or low-attached relative clauses. Sentences with a genitive case at the NP2 attachment site were used, with high-attachment prosody (e.g., Clifton et al., 2002), which had previously shown heterogeneous attachment preference across native German speakers and a greater comprehension difficulty compared to other attachment ambiguity constructions (Augurzky, 2006). Moreover, the previous experiments were unable to effectively analyze online comprehension of these sentence types, likely due to the heterogeneous preferences among participants. Our finding of a reliable offline source of online attachment preference can be employed by future studies. This may reveal comprehension processes among groups with heterogeneous attachment preference by focusing on preferred vs. non-preferred attachment as opposed to high vs. low attachment.

Independent of offline self-reported relative clause attachment preference, many participants used a pragmatic strategy to complete sentences. For example, these participants completed sentences based on the economics or practicality of whom they thought would visit a particular place, even if the answer sounded structurally less natural to them. This is consistent with previous studies that reported relative clause attachment to be influenced by pragmatic content (De Vincenzi and Job, 1995; Scheepers, 2003). The pragmatic strategy was reflected in the proportion of online answers that matched the offline reported preference, yet had no statistical bearing on the actual online attachment of relative clauses. Thus, future experiments should keep in mind the potential of pragmatics to bias relative clause attachment in stimuli; however, even in the admitted presence of a pragmatics strategy, its total influence on relative clause attachment can be negligible (Figure 2B).

The individual diagnostic scores that assessed working memory and musical ability had no relationship to relative clause attachment. This result supports a previous finding of no influence of working memory on visual relative clause attachment preference (Hocking, 2003) and casts further doubt on previous conflicting findings that working memory is linked conflictingly to low (Swets et al., 2007) and high (Traxler, 2007) attachment preference (Caplan and Waters, 2013; Van Dyke et al., 2014). While we found no role of working memory or musical rhythm ability in the preference for high or low relative clause attachment, we cannot distinguish from these findings whether these individual abilities play a role in the actual cognitive processing associated with resolution of syntactic ambiguities (e.g., Gunter et al., 2003; Bornkessel et al., 2004; Meyer et al., 2013) or the role that musical rhythm ability might play in language comprehension (Douglas and Willatts, 1994; Gordon et al., 2015a; cf. Swaminathan et al., 2018). Thus, future investigations may still find a relevant role of these individual differences in the online psycholinguistic processing of non-preferred relative clause attachment.

One potential explanation for why we did not find rhythm ability to correlate with attachment preference is that we controlled for disambiguating prosodic cues. We allowed our speaker to produce high-attachment prosody in terms of lengthening and comma phrasing; however, our cross-splicing rigorously eliminated any pitch or loudness cues that might have indicated an NP1 or NP2 attachment site (e.g., Schafer et al., 1996). While lengthening and natural pausing before commas falls under phonological processing, hypothesized here to be related to rhythm ability, so, too, do pitch and loudness (Nespor and Vogel, 1986). Perhaps listeners with better rhythm ability are better able to “tune in” to non-temporal phonological cues, which, in turn, may influence their online attachment preference. Therefore, we recommend future studies investigating the role of rhythm ability in attachment preference to include multiple types of prosodic cueing in their paradigm.

While neither working memory nor musical rhythm ability was linked to syntactic attachment preference itself, rhythm ability was linked to the presence or absence of a pragmatic strategy. Participants with higher musical ability were less influenced by pragmatic content and completed sentences more consistently with their own high or low syntactic preference. Our result aligns with a recent relative clause attachment priming paradigm in English, where participants with self-assessed high musical ability stuck to their syntactic preference for low attachment when primed for high attachment, whereas in the same context, participants with self-assessed low musical ability went against their low-attachment preference to attach high (Menon, 2018). Accordingly, a recent investigation of speech processing found that participants naturally divided into “high-synchronizers” and “low-synchronizers,” where magnetoencephalographic (MEG) data showed that high-synchronizers had increased brain-to-stimulus synchrony (Assaneo et al., 2019). Moreover, high-synchronizers had more musical training experience. In the context of our current results, perhaps participants who have greater rhythm ability are, in turn, better able to neurally synchronize to acoustic prosodic cues (as proposed in Tierney and Kraus, 2014). This would not necessarily have been reflected in the current study because of our controlled pitch and loudness prosodic cues. On the other hand, perhaps participants with less rhythm ability are less able to neurally synchronize to prosodic cues. These participants would have to rely on a different strategy all the time because they cannot ever “tune-in” to acoustic cues. Thus, our paradigm caught their strategy: the participants with poorer rhythm ability relied on world knowledge to disambiguate attachment ambiguities.

## Conclusion

The current study explored auditory relative clause attachment preference in monolingual native German speakers, potential underlying strategies for ambiguity resolution, and the possible roles of verbal working memory and musical rhythm skills. We found that attachment preference varied individually, but that offline, participants reliably reported their own online preferences. While working memory had no impact on attachment preference, we found that participants with poorer musical rhythm skills used a pragmatics-related ambiguity resolution strategy. Our finding that an offline self-report is sufficient to assign preference, allowing future psycholinguistic experiments access to previously occluded comprehension processes—in terms of preferred and non-preferred attachment, instead of high and low attachment—with a note that participants with lower musical skills might override their reported syntactic preference in favor of pragmatic context. An interesting direction for future research is the role of rhythm ability in auditory language comprehension strategy.

## Ethics Statement

This study was approved by the local ethics committee of the University of Leipzig and was carried out in accordance with the ethics guidelines of the University of Leipzig. All participants gave written informed consent in accordance with the Declaration of Helsinki.

## Author Contributions

EH, DS, and SK designed the study and wrote the manuscript. EH collected the data and performed analyses.

## Conflict of Interest Statement

The authors declare that the research was conducted in the absence of any commercial or financial relationships that could be construed as a potential conflict of interest.

## Footnotes

1 www.neurobs.com
