# Supplementary material for: Attachment Preference in Auditory German Sentences: Individual Differences and Pragmatic Strategy
Source: Front Psychol. 2019 Jun 18;10:1357. doi: 10.3389/fpsyg.2019.01357 (PMC6592159; doi:10.3389/fpsyg.2019.01357)
Supplement: Supplementary file 3 [file Data_Sheet_3.pdf]

1 Appendix 1 Stimuli

These sentence fragments were presented during the sentence completion experiment. Recordings had two versions (see Figure 1A), for a total of 240 auditory stimuli. The two versions were counterbalanced across participants, such that each participant heard these 120 lexical combinations once.

1. Da draußen plaudern die Brüder der Ärztin , die Salzburg vor kurzem
2. Dort drüben rasten die Brüder der Chefin , die Moskau vor kurzem
3. Da draußen schuften die Brüder der Türkin , die Kapstadt vor kurzem
4. Da draußen speisen die Brüder der Russin , die London vor kurzem
5. Dort vorne plaudern die Diener der Türkin , die Lübeck vor kurzem
6. Da draußen rasten die Diener der Witwe , die Basel vor kurzem
7. Dort drinnen schuften die Diener der Schwedin , die Brüssel vor kurzem
8. Da vorne speisen die Diener der Russin , die Moskau vor kurzem
9. Dort hinten plaudern die Freunde der Polin , die München vor kurzem
10. Dort vorne rasten die Freunde der Lettin , die Lübeck vor kurzem
11. Da hinten schuften die Freunde der Erbin , die Brüssel vor kurzem
12. Da drüben speisen die Freunde der Kundin , die London vor kurzem
13. Da vorne plaudern die Gäste der Lettin , die Moskau vor kurzem
14. Da drüben rasten die Gäste der Dänin , die Frankfurt vor kurzem
15. Dort draußen schuften die Gäste der Griechin , die Stuttgart vor kurzem
16. Da drinnen speisen die Gäste der Polin , die Kapstadt vor kurzem
17. Dort drinnen plaudern die Kumpel der Polin , die Salzburg vor kurzem
18. Da vorne rasten die Kumpel der Dänin , die Bregenz vor kurzem
19. Da draußen schuften die Kumpel der Griechin , die Frankfurt vor kurzem
20. Da hinten speisen die Kumpel der Britin , die München vor kurzem
21. Da draußen plaudern die Kunden der Finnin , die Warschau vor kurzem
22. Dort drüben rasten die Kunden der Britin , die Basel vor kurzem
23. Dort vorne schuften die Kunden der Griechin , die Bregenz vor kurzem
24. Dort draußen speisen die Kunden der Chefin , die München vor kurzem
25. Da hinten plaudern die Nachbarn der Dänin , die Patras vor kurzem
26. Da vorne rasten die Nachbarn der Schwedin , die Lübeck vor kurzem
27. Da drinnen schuften die Nachbarn der Britin , die Kapstadt vor kurzem
28. Dort vorne speisen die Nachbarn der Kundin , die Stuttgart vor kurzem
29. Dort draußen plaudern die Neffen der Britin , die Bregenz vor kurzem
30. Dort draußen rasten die Neffen der Finnin , die Nizza vor kurzem
31. Dort hinten schuften die Neffen der Erbin , die Warschau vor kurzem
32. Dort hinten speisen die Neffen der Dänin , die Ostrau vor kurzem
33. Da drinnen plaudern die Nichten der Polin , die Brüssel vor kurzem
34. Dort hinten rasten die Nichten der Russin , die Basel vor kurzem
35. Dort draußen schuften die Nichten der Schwedin , die Moskau vor kurzem
36. Dort vorne speisen die Nichten der Tschechin , die Stuttgart vor kurzem
37. Da drinnen plaudern die Onkel der Türkin , die Frankfurt vor kurzem
38. Da drinnen rasten die Onkel der Chefin , die London vor kurzem
39. Dort drinnen schuften die Onkel der Russin , die Nizza vor kurzem
40. Da hinten speisen die Onkel der Kundin , die Salzburg vor kurzem
41. Dort drinnen plaudern die Schwestern der Türkin , die Nizza vor kurzem
42. Da hinten rasten die Schwestern der Tschechin , die Warschau vor kurzem

43. Da vorne schuften die Schwestern der Erbin , die Ostrau vor kurzem
44. Da vorne speisen die Schwestern der Witwe , die Patras vor kurzem
45. Da drüben plaudern die Söhne der Chefin , die Warschau vor kurzem
46. Da hinten rasten die Söhne der Finnin , die Lübeck vor kurzem
47. Da drüben schuften die Söhne der Ärztin , die München vor kurzem
48. Dort drüben speisen die Söhne der Griechin , die Patras vor kurzem
49. Dort drüben plaudern die Tanten der Erbin , die Kapstadt vor kurzem
50. Dort drinnen rasten die Tanten der Lettin , die Basel vor kurzem
51. Dort drüben schuften die Tanten der Kundin , die Ostrau vor kurzem
52. Dort drüben speisen die Tanten der Schwedin , die Bregenz vor kurzem
53. Dort draußen plaudern die Töchter der Tschechin , die Nizza vor kurzem
54. Dort vorne rasten die Töchter der Ärztin , die Patras vor kurzem
55. Da drüben schuften die Töchter der Finnin , die London vor kurzem
56. Dort hinten speisen die Töchter der Witwe , die Salzburg vor kurzem
57. Da drüben plaudern die Vettern der Tschechin , die Ostrau vor kurzem
58. Dort hinten rasten die Vettern der Ärztin , die Stuttgart vor kurzem
59. Da drinnen schuften die Vettern der Lettin , die Brüssel vor kurzem
60. Dort drinnen speisen die Vettern der Witwe , die Frankfurt vor kurzem
61. Da draußen fachsimpeln die Brüder der Ärztin , die Wien vor kurzem
62. Dort drüben faulenzen die Brüder der Chefin , die Perm vor kurzem
63. Da draußen arbeiten die Brüder der Türkin , die Paarl vor kurzem
64. Da draußen frühstücken die Brüder der Russin , die Leeds vor kurzem
65. Dort vorne fachsimpeln die Diener der Türkin , die Trier vor kurzem
66. Da draußen faulenzen die Diener der Witwe , die Bern vor kurzem
67. Dort drinnen arbeiten die Diener der Schwedin , die Gent vor kurzem
68. Da vorne frühstücken die Diener der Russin , die Perm vor kurzem
69. Dort hinten fachsimpeln die Freunde der Polin , die Fürth vor kurzem
70. Dort vorne faulenzen die Freunde der Lettin , die Trier vor kurzem
71. Da hinten arbeiten die Freunde der Erbin , die Gent vor kurzem
72. Da drüben frühstücken die Freunde der Kundin , die Leeds vor kurzem
73. Da vorne fachsimpeln die Gäste der Lettin , die Perm vor kurzem
74. Da drüben faulenzen die Gäste der Dänin , die Mainz vor kurzem
75. Dort draußen arbeiten die Gäste der Griechin , die Ulm vor kurzem
76. Da drinnen frühstücken die Gäste der Polin , die Paarl vor kurzem
77. Dort drinnen fachsimpeln die Kumpel der Polin , die Wien vor kurzem
78. Da vorne faulenzen die Kumpel der Dänin , die Linz vor kurzem
79. Da draußen arbeiten die Kumpel der Griechin , die Mainz vor kurzem
80. Da hinten frühstücken die Kumpel der Britin , die Fürth vor kurzem
81. Da draußen fachsimpeln die Kunden der Finnin , die Lodz vor kurzem
82. Dort drüben faulenzen die Kunden der Britin , die Bern vor kurzem
83. Dort vorne arbeiten die Kunden der Griechin , die Linz vor kurzem
84. Dort draußen frühstücken die Kunden der Chefin , die Fürth vor kurzem
85. Da hinten fachsimpeln die Nachbarn der Dänin , die Kos vor kurzem
86. Da vorne faulenzen die Nachbarn der Schwedin , die Trier vor kurzem
87. Da drinnen arbeiten die Nachbarn der Britin , die Paarl vor kurzem
88. Dort vorne frühstücken die Nachbarn der Kundin , die Ulm vor kurzem
89. Dort draußen fachsimpeln die Neffen der Britin , die Linz vor kurzem
90. Dort draußen faulenzen die Neffen der Finnin , die Cannes vor kurzem

91. Dort hinten arbeiten die Neffen der Erbin , die Lodz vor kurzem
92. Dort hinten frühstücken die Neffen der Dänin , die Prag vor kurzem
93. Da drinnen fachsimpeln die Nichten der Polin , die Gent vor kurzem
94. Dort hinten faulenzen die Nichten der Russin , die Bern vor kurzem
95. Dort draußen arbeiten die Nichten der Schwedin , die Perm vor kurzem
96. Dort vorne frühstücken die Nichten der Tschechin , die Ulm vor kurzem
97. Da drinnen fachsimpeln die Onkel der Türkin , die Mainz vor kurzem
98. Da drinnen faulenzen die Onkel der Chefin , die Leeds vor kurzem
99. Dort drinnen arbeiten die Onkel der Russin , die Cannes vor kurzem
100. Da hinten frühstücken die Onkel der Kundin , die Wien vor kurzem
101. Dort drinnen fachsimpeln die Schwestern der Türkin , die Cannes vor kurzem
102. Da hinten faulenzen die Schwestern der Tschechin , die Lodz vor kurzem
103. Da vorne arbeiten die Schwestern der Erbin , die Prag vor kurzem
104. Da vorne frühstücken die Schwestern der Witwe , die Kos vor kurzem
105. Da drüben fachsimpeln die Söhne der Chefin , die Lodz vor kurzem
106. Da hinten faulenzen die Söhne der Finnin , die Trier vor kurzem
107. Da drüben arbeiten die Söhne der Ärztin , die Fürth vor kurzem
108. Dort drüben frühstücken die Söhne der Griechin , die Kos vor kurzem
109. Dort drüben fachsimpeln die Tanten der Erbin , die Paarl vor kurzem
110. Dort drinnen faulenzen die Tanten der Lettin , die Bern vor kurzem
111. Dort drüben arbeiten die Tanten der Kundin , die Prag vor kurzem
112. Dort drüben frühstücken die Tanten der Schwedin , die Linz vor kurzem
113. Dort draußen fachsimpeln die Töchter der Tschechin , die Cannes vor kurzem
114. Dort vorne faulenzen die Töchter der Ärztin , die Kos vor kurzem
115. Da drüben arbeiten die Töchter der Finnin , die Leeds vor kurzem
116. Dort hinten frühstücken die Töchter der Witwe , die Wien vor kurzem
117. Da drüben fachsimpeln die Vettern der Tschechin , die Prag vor kurzem
118. Dort hinten faulenzen die Vettern der Ärztin , die Ulm vor kurzem
119. Da drinnen arbeiten die Vettern der Lettin , die Gent vor kurzem
120. Dort drinnen frühstücken die Vettern der Witwe , die Mainz vor kurzem
